# Emerging Applications of Augmented and Mixed Reality Technologies in Motor Rehabilitation: A Scoping Review

**DOI:** 10.3390/s25072042

**Published:** 2025-03-25

**Authors:** Arman Farsi, Giacinto Luigi Cerone, Deborah Falla, Marco Gazzoni

**Affiliations:** 1 LISiN—Laboratory for Engineering of the Neuromuscular System, Department of Electronics and Telecommunications, Politecnico di Torino, 10129 Turin, Italy; arman.farsi@polito.it (A.F.); giacintoluigi.cerone@polito.it (G.L.C.); 2 PoliToBIOMed Lab, Politecnico di Torino, 10129 Turin, Italy; 3 Centre of Precision Rehabilitation for Spinal Pain (CPR Spine), School of Sport, Exercise and Rehabilitation Sciences, College of Life and Environmental Sciences, University of Birmingham, Birmingham B15 2TT, UK; d.falla@bham.ac.uk

**Keywords:** augmented reality, mixed reality, motor rehabilitation

## Abstract

Background: Augmented Reality (AR) and Mixed Reality (MR) are emerging technologies with notable potential for motor rehabilitation. Given the novelty and breadth of this field, this scoping review aims to identify how and to what extent AR and MR technologies are used in motor rehabilitation. Methods: We conducted a search in Scopus and PubMed (2010–2024), following PRISMA-ScR guidelines. In the analysis, we focused on four key aspects: (I) the AR/MR display technologies, (II) the sensors used to collect data to generate the augmented information, (III) the pathologies addressed, and (IV) the assessment of usability and acceptability. Results: Among 105 selected studies, 58% developed new prototypes, while 42% tested existing systems. Head-mounted displays were the most common device (56.2%), followed by monitors (34.3%) and video projectors (14.3%). The most commonly used sensors were RGB-D cameras (31.4%), sensors for localization and mapping (33.3%), normal cameras (17.1%), and electromyography sensors (14.3%). Regarding the target pathology, 34.2% of studies did not focus on a specific pathology, 26.7% were on stroke, 10.5% on limb loss, and 9.5% on Parkinson’s disease. Over half (51.4%) of the studies investigated usability and acceptance. Conclusions: AR/MR technologies hold promise for motor rehabilitation, but limited comparative studies and long-term investigations currently hinder a clear understanding of their benefits.

## 1. Introduction

Motor rehabilitation aims to restore motor functional capabilities to improve the quality of life of patients [1]. Traditional rehabilitation approaches involve one-on-one therapy sessions with a physical therapist or occupational therapist; these sessions commonly target range of motion, strength, coordination, and balance, aiming to help patients regain motor function.

The introduction of technologies in motor rehabilitation has fostered a data-driven approach, enabling the tracking of progress, the adjustment of treatment plans, and the implementation of more informed decision-making. New technologies such as wearable devices, telerehabilitation, and serious games have improved continuous monitoring, access to therapy, and patient engagement, respectively. Among others, in recent years, extended reality technologies have made significant progress and are now mature enough to be applied in many fields, including motor rehabilitation.

Extended Reality (XR) is an umbrella term encompassing Augmented Reality (AR), Mixed Reality (MR), and Virtual Reality (VR), which represent a spectrum of technologies that merge physical and digital worlds [2,3]. On one end of the spectrum, VR creates a fully immersive digital experience, shutting out the physical world. On the other end, AR shows digital information within the user’s environment in real-time [4]. MR lies in between, merging real and virtual worlds to produce new environments where physical and digital objects co-exist and interact [5].

XR technologies are of particular interest in the field of rehabilitation since they allow: (I) interactive environments to be created to engage patients during their rehabilitation, aiming to improve patient motivation and adherence to therapy, (II) tailoring of exercises to individual patient needs and abilities and (III) implementing a great variety of scenarios for example, retraining activities of daily living.

Despite the promising potential of XR technologies in motor rehabilitation, the use of these technologies for rehabilitation purposes has not yet been fully explored. The literature on this topic is growing, but it remains scattered and diverse, with studies employing different technologies, methodologies, and outcome measures. Therefore, there is a need to map the existing literature to identify gaps in knowledge, which will guide future research.

The main objective of this scoping review was to identify how and to what extent AR and MR technologies are used in motor rehabilitation. Given the novelty and breadth of this field, a scoping review approach was deemed as the most appropriate to understand the extent and nature of the research. This work could serve as a preliminary step towards a Systematic Review in future research.

We specifically examined four key aspects: (I) the different AR/MR display technologies currently employed, (II) the sensors used to obtain data to generate the augmented information delivered to patients, (III) the pathologies addressed, and (IV) the assessment of usability and acceptability.

## 2. Methods

This scoping review was conducted following the PRISMA extension for scoping reviews (PRISMA-ScR) [6], but no protocol was registered for this scoping review. The review followed five iterative steps recommended for scoping reviews: (a) identifying the research question, (b) searching for relevant studies, (c) selecting studies, (d) charting the data, and (e) collating, summarizing, and reporting the results [7,8].

### 2.1. Eligibility Criteria

To be considered for inclusion in this review, the paper needed to address the use of AR/MR in motor rehabilitation as a primary intervention or as a tool for assessment or training. The papers were required to be published between 2010 and 2024 and written in English. The year 2010 was selected as the starting point due to a significant surge in publications from that year onwards, as numerous industries began to implement AR/MR technologies [9].

Exclusion criteria primarily focused on VR therapeutic approaches, psychological or psychiatric applications of AR/MR technologies, or their cognitive or emotional effects. Articles discussing applications in medical fields other than motor rehabilitation (e.g., surgical, diagnostic, dental, cardiovascular, and others) were also excluded.

### 2.2. Information Sources

To identify relevant studies for this scoping review, a comprehensive search was conducted in June 2024 using the Scopus and PubMed databases. In December 2024, two authors (AF and MG) conducted a literature search for the latest published works and updated the list of papers accordingly using the Scopus and PubMed databases.

### 2.3. Search Strategy

The search strategy was defined by combining some terms defining the technologies of interest (“mixed reality”, “augmented reality”, “HoloLens”) together with the application field (“rehabilitation”).

The terms identifying the technology were combined with the Boolean operator “OR”, while the technology and the application field were combined with the Boolean operator “AND”. The search for keywords was limited to the “Title”, “Abstract”, and “Keywords” sections of the articles. The English language was used as a limit to filter the documents. The search was limited to regular papers, conference papers, and reviews published in journals and conference proceedings between 2010 and 2024.

The details of the queries used in Scopus and PubMed are reported in Appendix A.

### 2.4. Selection of Sources of Evidence

Titles, abstracts, and full text of the publications were evaluated by two authors (AF, MG) using Rayyan (Rayyan Systems Inc., Cambridge, MA, USA) [10], a free web tool for systematic reviews. Independent screening was conducted by each reviewer, and conflicts were resolved through discussion until an agreement was reached. Initially, the two reviewers independently examined 50% of the records by title and abstract. Any discordance was addressed through discussion, relying on pre-established eligibility criteria to ensure a consistent screening process. Following the complete examination of all records, an additional meeting was held to mitigate any inconsistencies among the reviewers.

Records that were questionable in terms of their eligibility for inclusion in the review were selected for full-text analysis. During the full-text review process, we excluded conference papers that had been extended to full papers, study protocols, preliminary or work-in-progress records, review papers, and papers lacking full-text availability.

### 2.5. Data Charting

Following the evaluation and selection of the studies, a data-charting form was jointly developed in Excel by AF and MG to determine which variables to extract. The data-charting form was tested on a subset (20%) of the eligible papers and refined. Data charting was performed independently by the two reviewers. Any disagreements were settled by discussion between the two reviewers.

### 2.6. Data Items

The data charted from the studies encompassed the following items: the author’s name, the publication year, the “AR/MR display device” used, the “source of information in AR/MR”, the “pathology” the system was developed for, the “anatomical region” the rehabilitation tool was focused on, the “number of participants” the system was tested on, and the “acceptability and usability analysis” used within the study, if any.

The “AR/MR display device” item encompasses three main categories: (I) Head-mounted display (HMD), (II) Monitor, and (III) Video projector. The HMD category includes see-through headsets that overlay digital images in a real-world environment. In this category, we also included VR-HDM using external cameras to show the real environment to the user. The Monitor category includes external displays. The Video projector category includes all devices that project virtual cues in the real environment.

The “Source of Information in AR/MR” item encompasses the sensors employed to acquire the data used to add digital information to the real world (e.g., cameras, kinematic and kinetic sensors, and equipment for measuring physiological signals). Among them, we included two integrated systems: the HTC Vive Tracker and the Simultaneous Localization and Mapping (SLAM) systems. The HTC Vive Tracker is a widely used commercial system for full-body and/or object tracking. The SLAM systems map the environment and estimate the position of a subject/object within it at the same time [11,12]. Since the sensors in the HoloLens enable SLAM, if the device is used not only for visualization but also for SLAM, we refer to them collectively as ’SLAM’ instead of listing each sensor individually.

### 2.7. Synthesis of Results

Quantitative data were summarized using frequency counts and descriptive statistics, while qualitative data were analyzed thematically to identify recurring concepts and trends. The findings are presented in a combination of narrative synthesis, tabular summaries, and visual representations such as pie charts and percentage distributions, emphasizing the relevance of each category.

## 3. Results

### 3.1. Selection of Sources of Evidence

**Figure 1:**
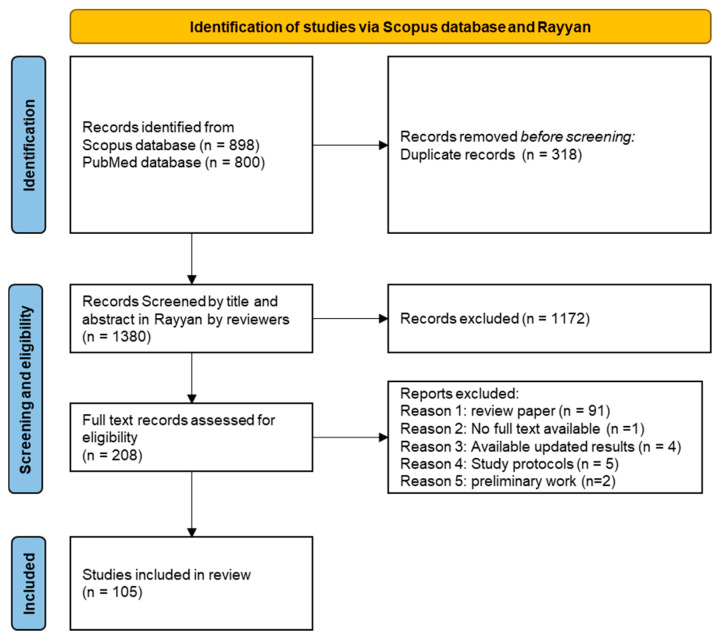
Flow diagram depicting the study selection process.

From an initial pool of 1698 records, by searching the specified keywords in Scopus and PubMed databases, a total of 1380 papers were selected, applying the filters on the publication date, document type, and language. After screening of the abstracts, 1172 papers were excluded. During the full-text review, among the remaining 208 records, we excluded 103 papers which were not considered to be original quantitative research (e.g., review papers), 4 papers [13,14,15,16] due to being preliminary work later fully developed into a journal article, 2 papers presenting only preliminary findings with the prospect of future research [17,18], 5 papers outlining the protocol for controlled trials [19,20,21,22,23,24], and 1 paper that was not accessible. Finally, we deemed 105 studies eligible for inclusion in this scoping review, as shown in the PRISMA flow diagram (Figure 1).

### 3.2. Characteristics of Sources of Evidence

**Figure 2:**
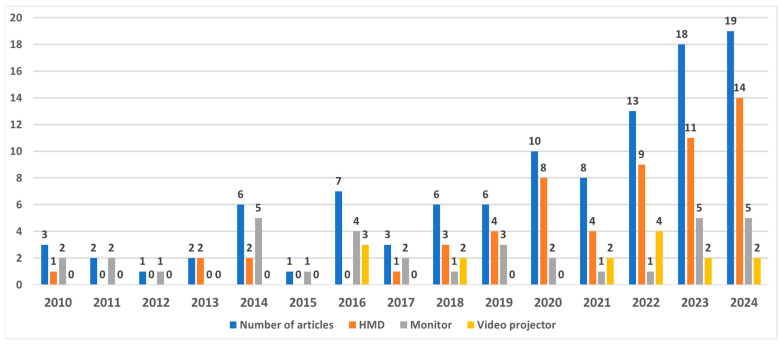
Trends in the Use of Display Devices in AR/MR Studies Over Time. There is an increase in the number of publications from 2010 to 2024, indicating a growing interest in the application of AR/MR within the field of motor rehabilitation.

Upon analyzing the publication dates of the records (Figure 2), it was observed that there was a substantial increase in the number of publications from 2010 (3 publications) to 2024 (19 publications). This trend indicates a growing interest in the application of AR/MR within the field of motor rehabilitation.

Of the 105 studies that met the inclusion criteria, all focused on the development and/or validation of an AR/MR system in motor rehabilitation. Among these, 58% of the papers focused on the development of a concept/prototype to apply AR/MR in motor rehabilitation and 42% on the application of a prototype in a clinical trial.

Most studies involved a small number of participants (median: 18, first quartile Q1: 5.5, third quartile Q3: 25). Only a few studies deviated from this trend, notably four with sample sizes of 56 [25], 81 [26],115 [27] and 120 participants [28].

Among these studies, 35% focused on patients, 43% on healthy participants, 10% on a mix of both, and 11% were either proof of concept or did not state the number of participants.

### 3.3. Results of Individual Sources of Evidence

Table 1 reports the list of all sources of evidence together with the relevant data that were charted in relation to the review questions and objectives.

### 3.4. Synthesis of the Results

In the subsequent sections, we present a comprehensive analysis for each specified item, encompassing aspects such as AR/MR display device preferences, sources of information utilized in AR systems, and the anatomical focus and pathological conditions targeted in AR/MR-based rehabilitation studies. This analysis further delves into usability and acceptability assessments, emphasizing the methodological tools employed and user acceptance metrics.

#### 3.4.1. AR/MR Display Device

HMDs, employed in 56.2% of the reviewed studies, were the most popular choice to provide AR/MR information to the users. Monitor systems were used in 34.3% of the studies, and video projectors in 14.3%. Some studies used more than one display type, which can push the total percentage over 100%. As illustrated in Figure 2, the evolution of display device usage from 2010 to 2024 highlights a pronounced uptake in the use of HDMs starting in 2022. By 2024, HMDs dominate, appearing in about 74% of the reviewed studies (14 out of 19). Monitors remain a secondary choice at 26% (5 out of 19), followed by video projectors at 11% (2 out of 19). This surge in HMD usage underscores the growing preference for immersive hardware solutions, even as monitors and projectors continue to provide viable alternatives in a smaller share of investigations.

#### 3.4.2. Source and Conveyed Information

The landscape of sensor technology utilized to collect the data needed to generate augmented information in AR/MR systems is diverse. The most commonly used sensors were SLAM (33.3%), RGB-D cameras (31.4%), normal cameras (17.1%), electromyography (EMG) sensors (14.3%), force platforms (12.4%), Inertial Measurement Units (IMU) (11.4%) and infrared cameras (11.4%).

#### 3.4.3. Pathology and Anatomical Region

**Figure 3:**
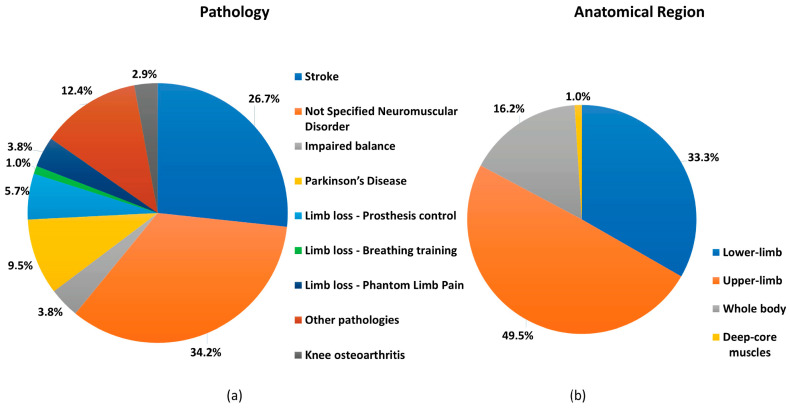
(**a**) Pie chart illustrating the pathologies addressed in the analyzed studies. The majority of the selected records (34.2%) focused on developing AR/MR systems without targeting specific neuromuscular disorders, while stroke was the most frequently targeted pathology, representing 27% of the studies. (**b**) Pie chart illustrating the anatomical regions targeted by AR/MR rehabilitation devices in the selected studies. Notably, half of the records focused on the upper limb, highlighting the emphasis on this body region.

Most of the studies (34.2%) focused on the development of an AR/MR system without a specific neuromuscular disorder as a target; hence, we considered them applicable to a wide range of pathologies. The studies which considered a specific disorder/disabling condition focused on Stroke (26.7%), Limb loss (10.5%), Parkinson’s disease (9.5%), Impaired balance (3.8%), and Knee osteoarthritis (2.9%). A few studies (11.4%), each singular in their focus, targeted conditions such as Hereditary Spastic Paraplegia, Cerebral Palsy, Cerebellar Ataxic, Adolescent idiopathic scoliosis, Rotator cuff tear, Ankle sprain, Sarcopenia, Developmental Coordination Disorder, Anterior Cruciate Ligament, Multiple Sclerosis, Pelvic Floor Dysfunction and Tremor (Figure 3a).

The “upper limb” was the most frequent anatomical region of focus (49.5%), followed by the “lower limb” (33.3%) and “whole body” (16.2%). One study was not included within these three main categories since it focused on deep core muscles (Figure 3b).

#### 3.4.4. Non-Specified Neuromuscular Disorders

Among the papers without a specific neuromuscular disorder as a target, the majority (20 papers) focused on upper limb [31,36,39,40,55,61,92,97,108,120,125] or hand [42,83,87,94,109,110,116,117] movement rehabilitation; ten studies [32,50,66,75,85,89,90,98,105,111] focused on gait retraining.

In most of the studies (29/37), AR/MR was used to provide patients with visual cues to follow, grasp, or reach, as well as obstacles to avoid. In five [90,94,96,102,111], a virtual avatar was shown to guide the patients in the correct execution of rehabilitation exercises. In another [45], the skeletal structures and the joint angles were rendered as a holographic overlay on the patient, as if the user had “X-ray vision”. In one [48], a set of metrics and data representing the quality of the current exercise were provided in real time to the therapist through holograms projected all around the patient. A single study focused on the real-time feedback of muscle activation, showing a color map overlayed on the muscle of interest [4].

#### 3.4.5. Stroke Rehabilitation

Most of the studies (68%) focused on upper limb rehabilitation, implementing AR/MR systems for occupational therapy to improve gross and fine motor skills in everyday tasks [30,35,37,38,41,43,44,46,51,56,59,63,64,70,79,91,104,124,128]. Approximately 18% of the studies were focused on lower limb rehabilitation, in particular on gait retraining [47,62,69,72,115]. Meanwhile, 15% were focused on the whole body using different rehabilitation strategies for motor recovery to impact functional disability [73,74,113,126].

#### 3.4.6. Limb Loss

Among the papers focused on limb loss, we distinguished the type of intervention in Prosthesis Control, Phantom Limb Pain (PLP) treatment, and Breathing training.

- Prosthesis Control: Five papers explored the use of AR/MR technology to enhance prosthetic limb control. Four papers [60,65,87,106] focused on upper limb prosthetics, and two [80,107] on the lower limb. Four studies [65,80,106,107] utilized HMDs to show the subject a virtual limb whose movement was controlled by the EMG signals from the residual muscles. One paper [60] used AR glasses to show a command window to control a virtual prosthesis. The interaction with the command window was realized by tracking the user’s head position. The user simultaneously received AR visual feedback about the command and, for example, grip strength or the degree of hand closure.
- Phantom Limb Pain: Phantom limb pain is a form of neuropathic pain experienced in the region of a missing limb. Four records [26,95,100,114] proposed AR/MR as a tool to reduce phantom limb pain by displaying a virtual arm attached to the residual limb and controlled by EMG.
- Breathing Training: In one study [77], an AR headset was used to provide lower limb amputees with feedback about deep core muscle activity and thoracic excursion during breathing training for back pain reduction.

#### 3.4.7. Parkinson’s Disease

In Parkinson’s disease, the main focus of rehabilitation is usually on gait training, management of freezing events, and balance improvement. In Parkinson’s disease, the step length is usually shortened, and walking speed is reduced. In all studies [29,34,49,58,67,99,101,121,122], visual cues were provided to the patients as targets in different scenarios to improve gait, except one study [86], which focused on upper limb rehabilitation, improving hand–eye coordination in 3D space by integrating MR and Haptic Device. In one study [29], smartglasses’ IMU sensors were used for the real-time detection of Freezing of Gait (FOG) events. When a FOG event is detected, the system creates visual guidance for the patient, seen through the glasses as targets on the floor. In another study [67], researchers evaluated whether AR visual cues improve turning in patients with Parkinson’s disease. The system shows a series of small spheres equally spaced at a semicircle around the participant who is requested to “eat” them with a virtual sphere shown in front of them. In other studies [34,49,58,99,101,122,125], patients were requested to walk along a simple path showing virtual targets to step on or catch, as well as obstacles to avoid.

#### 3.4.8. Impaired Balance

Four studies focused on postural instability, exploring how AR interventions could aid in improving postural control in a secure setting. In one study [33], AR was used to show a ball hologram that subjects had to interact with to stimulate sideways motion and rotations of the upper body. In another [78], AR was used to provide simple feedback on head movement to the subject, and they were requested to minimize movement during stance tasks. Two studies [28,103] explored the use of HOLOBalance and HoloBox systems, a virtual coach in AR, to guide patients through balance exercises.

#### 3.4.9. Knee Osteoarthritis

Three studies [25,71,127] investigated the use of AR/MR technologies in the rehabilitation of knee osteoarthritis. AR/MR was used to guide users using either a visual guide, an avatar, or written summaries about how to perform the movements.

#### 3.4.10. Other Pathologies

- Hereditary Spastic Paraplegia [119] and Developmental Coordination Disorder [76]: a C-Mill treadmill was used to display visual cues for guiding steps or avoiding obstacles.
- Cerebral Palsy [57]: visual cues indicative of walking speed were shown to the patient via smartglasses to improve walking capabilities.
- Adolescent Idiopathic Scoliosis [93] and Rotator Cuff Tear [27]: these studies employed sensors to assess the participants’ postures and utilized monitors to communicate exercise instructions and provide feedback for correcting their movements.
- Tremor [123] and sarcopenia [68]: a virtual coach, manifested through a hologram avatar displayed on an HMD, was used to guide individuals through tailored exercise programs.
- Cerebellar Ataxia [53]: two exergames were developed focusing on upper limb coordination. In the first game, participants had to move a virtual spaceship to different planets. The second game required following the spaceship’s square path with their hand, guided by a 3D wormhole visual that helps align hand-eye coordination, aiming to follow the wormhole’s central axis directly to the target.
- Ankle sprain [54]: an exergame for mobile devices was developed focusing on the lower limb, using Mobile Augmented Reality to deliver a range of motion exercises as well as monitor the user’s performance. In this game, the subject is seated with the edge of the heel on the floor whilst holding a mobile device and is instructed to pivot and mimic the foot based on the virtual cues displayed on the screen.
- Anterior Cruciate Ligament Reconstruction [81]: an AR-based telerehabilitation system was developed for patients recovering from Anterior Cruciate Ligament Reconstruction. The system provided real-time feedback and exercise tracking through a 3D motion capture camera, allowing patients to perform rehabilitation at home. A randomized controlled trial showed similar functional improvements to conventional rehabilitation, with faster quadriceps strength recovery in the AR group.
- Multiple Sclerosis [112]: an exergame was developed for individuals with Multiple Sclerosis to support upper limb rehabilitation. The game integrates bimanual tasks, requiring users to manipulate real objects while balancing virtual elements, aiming to improve motor coordination and functional abilities. Initial tests with healthy participants demonstrated feasibility, with future trials planned for patients with Multiple Sclerosis to assess therapeutic benefits.
- Pelvic Floor Dysfunction [84]: an exergame was developed for older women with pelvic floor dysfunction, incorporating platform-jumping mechanics and real-time motion tracking to support Pelvic Floor Dysfunction rehabilitation. The system provides interactive feedback to enhance motivation and adherence.

### 3.5. Usability and Acceptability Assessment

Among the 105 selected studies, 54 examined the acceptability and usability of the proposed AR/MR systems. The primary methodology was the use of questionnaires in 48 out of 54 studies.

Regarding acceptability and usability research, questionnaires often necessitate a standardized rating scale to quantify responses for statistical analysis. Among the selected records, the Likert scale was the predominant choice for this purpose [26,31,34,36,38,39,40,43,44,47,50,62,66,68,73,76,77,94,97,98,101,104,105,108,111,112,113,120,123,126].

For the specific assessment of system usability and acceptability, the System Usability Scale (SUS) emerged as the frontrunner, featuring in 15% of the methods employed [31,36,38,43,50,64,98,101], with scores generally falling in the moderate to high range. Additional methodologies included the Intrinsic Motivation Inventory, tapping into the motivational aspects of engagement [38]; the Physical Activity Enjoyment Scale (PACES), assessing the enjoyment participants gather from the physical activities in the interventions, highlighting how these elements contribute to their overall pleasure [47]; and the User Experience Questionnaire, shedding light on broader aspects of user interaction [57,68,76,94,102,118,126]; the Pittsburgh Rehabilitation Participation Scale (PRPS) [66], which measures patient engagement levels; the User Satisfaction Evaluation Questionnaire [79], offering a direct measure of contentment with the system; the NASA-TLX Subjective Assessment Questionnaire [85,86], which gauges perceived workload; and the Self-reported Subjective Effort Questionnaire (SEQ) [77], assessing the effort expended by users during the rehabilitation process.

## 4. Discussion

This scoping review focused on the period 2010–2024. Early foundational research in the period 2000–2010 played a crucial role in shaping AR and MR applications in motor rehabilitation. However, AR/MR technologies became significantly more feasible and widely adopted after 2010, with the introduction of new technologies for motion tracking (e.g., Microsoft Kinect (2010; Microsoft Corporation, Redmond, WA, USA) and Leap Motion (2013; Leap Motion, Inc., San Francisco, CA, USA). From 2016 onward, the integration of wearable sensors and smart glasses (e.g., Microsoft HoloLens) further expanded AR/MR applications for the treatment of neuromuscular disorders.

We identified 105 studies that applied AR/MR technologies to a variety of motor rehabilitation interventions. All papers focused on developing/evaluating prototypes with a variety of methodological approaches. Usually, the number of participants the systems were tested on was limited, and no long-term field trials existed. Only a few studies compared AR/MR-based treatments with distinct approaches [91,92,103,125].

### 4.1. How Is AR/MR Used in Rehabilitation?

In the reviewed papers, AR/MR technologies are usually used to show virtual objects in the real environment to guide a patient’s movement during exercises. In gait rehabilitation, AR/MR has been widely employed to display virtual footprints which serve as navigational aids that patients must follow to enhance/train their walking patterns [28,29,32,47,69,72,76,99,101,105,122]; to further challenge and develop patient’s mobility and cognitive planning, virtual obstacles are generated, requiring users to maneuver around them [62,66,75,85,115,121]. In other studies, virtual objects/avatars were rendered within the patient’s environment to guide them to achieve a specific level of performance during the required task in terms of, for example, velocity and step length [50,57,68,71,89,90,98,102,111].

In the context of upper limb rehabilitation, AR/MR is usually used to show virtual objects with which the patient must interact. These can vary from simple visual cues that trigger interaction [35,38,40,42,43,44,53,59,61,63,64,82,86,92,97,104,109,110,112,117,124,125] to virtual prosthetic limbs that the user has to control [26,55,60,65,95,100,106,114]. Some applications use AR/MR to guide patient movements along pre-set virtual trajectories [39,51,56,79,83,91,108,120], while others show virtual trainers or avatars to demonstrate and assist with the execution of tasks [27,31,36,41,93,94].

### 4.2. AR/MR Display Technologies

AR/MR scenarios are created using different display technologies. HMDs are at the forefront in the display domain within this field, representing 56.2% of total usage (with Microsoft HoloLens glasses emerging as the most popular smart glasses with 64% of HMDs). Traditional monitor systems still hold a significant stake, accounting for 34.3%, while video projector systems account for only 14.3%.

Among the three technologies, HDMs allow a wide range of movement, an immersive AR/MR experience, and personalization of the experience. HMDs equipped with the SLAM feature can map the environment and track the position of users without the need for external devices. However, HMDs suffer from some challenges related to optical and display technology. Among these challenges, there is the Vergence–Accommodation Conflict (VAC) which causes visual discomfort and fatigue during prolonged use [129], the limited Field of View (FOV), which remains significantly narrower than natural human vision (35° in HoloLens1 [130], 52° in HoloLens 2 versus ~200° in human vision), luminance/contrast [39], image registration accuracy, depth perception, latency [60], and encumbrance. While these issues are more critical in applications, such as AR/MR-assisted surgery, they remain largely unexplored in rehabilitation, lacking both standardized evaluation methods and established benchmarks. Typically, they are evaluated in terms of user experience, including comfort, fatigue, and ease of interaction, rather than direct rehabilitation outcomes. Currently, there are no quantitative data linking the optical/display challenges to specific rehabilitation outcomes such as motor recovery improvements or therapy effectiveness. Their impact on the development and application of AR/MR systems in rehabilitation should be a topic of investigation.

Video projector systems have some advantages with respect to HDMs in terms of cost, comfort (HMDs are usually bulky and heavy), the possibility to share the experience with multiple users at the same time, and no limitations in the field of view. They are extensively used to project visual cues in the environment [50,75].

Even if traditional monitors provide a less immersive experience with respect to the other technologies, their simplicity, widespread availability, and the familiarity that many users have with them can make them less intimidating and more approachable, particularly for individuals who may find the complexity of more immersive systems daunting.

The choice between these systems typically depends on the specific requirements of the application and user preferences rather than a clear-cut advantage of one technology over others. Traditional monitor systems still hold a significant share but may be gradually overtaken as technology evolves and the benefits of more immersive experiences become evident.

### 4.3. Sensors Used to Add Augmented Information to the Real World

In 90% of the reviewed papers, the kinematics of patient movement was measured to create visual cues and objects and to allow interaction with them. Kinematics were captured using external sensors such as RGB-D cameras, IMU sensors, and infrared cameras or using the sensors integrated within the HMD itself for tracking human motion, thereby enabling the HMD to serve both as a tracking sensor and a feedback device.

In only 15 studies, electrophysiological signals were harnessed as a source of data. Among these, EMG signals were utilized in most [4,26,30,41,65,70,80,87,95,100,106,107,114]. In most studies [26,65,81,87,95,100,106,107,114], EMG was used to control a virtual limb. In one study [4], EMG was used to provide feedback about muscle activation. In another [77], plethysmographic signals were employed for training in pursed-lip breathing techniques.

### 4.4. Target Pathology

AR/MR technologies are being applied in a wide spectrum of medical conditions, addressing both generalized and specific neuromuscular disorders. The most common approach is to develop general-purpose systems without targeting a specific pathology. For some pathologies with distinctive characteristics and specific aspects to treat, ad-hoc systems were developed. For example, research has focused on conditions like Parkinson’s disease and limb loss, employing AR/MR technologies to address unique challenges, from predicting FOG to enhancing prosthesis control and alleviating phantom limb pain.

The widespread applicability and adaptability of AR/MR technologies highlight their potential to reshape therapeutic approaches and offer innovative and tailored solutions for a variety of neuromuscular disorders.

### 4.5. Assessing Usability and Acceptance

Usability and acceptability form crucial pillars in the success of any technological intervention as a preliminary step before evaluating treatment effectiveness. With a substantial number of AR/MR-based studies emphasizing these aspects, it is evident that understanding and improving the user experience remains a priority. The usability and acceptability of the technology was assessed in 53.3% of the selected studies. Encouragingly, positive feedback from many of the implementations underscores the potential and acceptability of AR/MR in the rehabilitation realm.

Despite some open challenges, the usability of AR systems is improving, and the cost of AR systems is expected to decrease as technology becomes more widespread, thanks to industrial and entertainment applications. As the technology continues to develop, we can expect to see more user-friendly and affordable AR systems that can be used for motor rehabilitation.

### 4.6. Further Considerations

Comparative analysis between AR/MR and conventional rehabilitation methods is infrequent, with only a handful of studies (4 records) addressing this aspect. For instance, one study [91] compared AR-based rehabilitation using a video projector with a traditional PC-based setup in post-stroke patients. The AR environment, which allowed direct interaction with virtual objects, demonstrated superior motor performance, leading to 21% higher game scores, 19% faster reaching times, and 15% less movement variability compared to the PC-based system. These findings suggest that AR enhances motor learning, spatial alignment, and patient engagement due to its direct visuomotor integration and reduced cognitive load. However, challenges such as technical complexity and limited accessibility highlight the need for further comparative research to assess its long-term clinical efficacy and real-world applicability. A comparative study [92] examined MR rehabilitation using the HoloLens 2 versus robotic-assisted therapy with the NRC end-effector-based rehabilitation arm (NREH) in reaching exercises. The results showed that robotic therapy significantly outperformed MR rehabilitation in execution speed (26–62% shorter completion time), movement precision (22–65% straighter trajectories), and reaching velocity. However, MR-based rehabilitation provided greater movement freedom, three-dimensional interaction, and higher cognitive engagement, suggesting that robotic therapy is better suited for early-stage recovery, while MR may be more beneficial for later-stage, independent home-based training. The study emphasizes the potential for hybrid rehabilitation models that combine structured robotic assistance with MR-driven interactive therapy. Another comparative study [125] analyzed the differences in motor performance and cognitive load when using immersive virtual reality (IVR), AR, and a standard 2D screen for goal-directed reaching movements. IVR demonstrated the best movement efficiency, with significantly faster reaction times, smoother movement trajectories, and fewer corrections than both AR and 2D screens. AR showed a slight performance advantage over 2D screens, but its effectiveness was limited by restricted FOV and minor tracking misalignments. Notably, cognitive load remained similar across all conditions, indicating that motor performance is more influenced by display modality than cognitive effort. These findings suggest that IVR is optimal for immersive motor training, while AR may be better suited for functional task training. Lastly, a study [105] compared projection-based AR with traditional monitor-based visual feedback for gait rehabilitation. Projection-based AR resulted in significantly faster gait adaptation (66% faster for stepping, 85% faster for obstacle avoidance), improved spatial accuracy (normalized accumulative deviation: 735.9 in AR vs. 884.1 in monitor-based feedback), and better real-time interaction. AR also eliminated visuospatial transformation errors, allowing for more natural movement adjustments. However, challenges such as occlusion handling and projection distortion must be addressed to enhance its clinical applicability. The findings indicate that hybrid rehabilitation approaches, combining projection-based AR with structured training, could improve gait retraining outcomes.

The scarcity of comparative studies limits the ability to assess the relative benefits and limitations of AR/MR technologies in rehabilitation. While preliminary evidence suggests that AR/MR can enhance motor learning, engagement, and real-time interaction, further research is needed to evaluate long-term functional outcomes and compare AR/MR to conventional therapist-guided rehabilitation.

### 4.7. Future Directions in AR/MR Rehabilitation

This scoping review has highlighted the growing application of AR/MR technologies in motor rehabilitation. Building upon the current trends and identified gaps, future research should prioritize the following areas to further advance this field:

1. Enhanced Treatment Assessment and Outcome Measures:
  - Conduct rigorous comparative studies to evaluate the efficacy of AR/MR-based rehabilitation compared to traditional rehabilitation methods.
  - Investigate the use of AR/MR in long-term rehabilitation treatments.
  - Investigate the cost-effectiveness of AR/MR interventions in different clinical settings and patient populations.

2. Expanding Research into Specific Populations and Conditions:
  - Extend studies to explore the application of AR/MR in the rehabilitation of specific patient populations and to address specific rehabilitation challenges, such as training of gait and upper limb function.

3. Addressing Medical device requirements, Visualization, and User Experience Challenges:
  - Critically assess the suitability of off-the-shelf, consumer-grade hardware and commercial game engines for medical applications, given the stringent requirements and standards for medical devices.
  - Develop innovative AR/MR display technologies to address current challenges and mitigate adverse effects associated with prolonged AR/MR use. This includes addressing limitations in the field of view, the Vergence–Accommodation conflict, the limited luminance/contrast, image registration accuracy, latency, and encumbrance.

By focusing on these key areas, future research can further establish the clinical effectiveness and practical utility of AR/MR technologies in motor rehabilitation, ultimately improving patient outcomes and quality of life.

## 5. Strengths and Limitations

This review scrutinized publications in the literature starting from 2010 to 2024, aiming to explore the application of AR/MR technologies for motor rehabilitation. We followed a transparent, rigorous, and standardized method throughout the scoping review process. All references were screened in all phases by two independent reviewers who communicated regularly to resolve conflicts.

The literature review is based on only two databases; however, we believe that the combined use of Scopus and PubMed provides a balanced approach that captures both the technological and clinical aspects of AR/MR in motor rehabilitation. Scopus ensures a broad coverage of engineering and technological developments, while PubMed focuses on clinical applications and medical evidence.

Identifying the most fitting search keywords presented a significant hurdle due to the specific focus of this review. The spectrum of terms and definitions within the mixed-reality domain varies widely, complicating the task. The scope of the review was particularly challenging as it concentrated on the use of MR/AR technologies in motor rehabilitation—a broad domain. The authors devised and tested a search strategy to capture as many relevant studies as possible from the selected database. The initial strategy, which included terms such as “neuromuscular rehabilitation” and “motor rehabilitation,” led to the exclusion of pertinent studies. Consequently, the term “rehabilitation” was chosen for its broader inclusion criteria. The process of selecting studies that aligned with the review’s goals was meticulously carried out during the abstract and full-text review phases.

## 6. Conclusions

A scoping review was conducted to identify and synthesize the literature on the use of AR/MR in motor rehabilitation. While AR/MR applications in motor rehabilitation are still in their early stages, their potential to enhance therapy through immersive, interactive, and personalized experiences is emerging. However, several limitations impede a full evaluation of the adoption of AR in motor rehabilitation; the lack of comparative studies with traditional rehabilitation approaches and the scarcity of long-term studies limit the evidence base for AR’s true advantages and effectiveness. Moreover, the cost associated with AR technology and equipment poses a financial barrier in healthcare settings.

For AR/MR to transition from experimental use to routine clinical practice, future efforts should focus on developing more affordable and user-friendly AR systems, integrating AR solutions with conventional rehabilitation programs, integrating AR into existing clinical workflows, and conducting rigorous comparative studies to establish its true benefits. Future research should prioritize these areas to bridge the gap between technological potential and real-world implementation, ensuring AR/MR transition from an emerging innovation to a practical tool that enhances rehabilitation outcomes in diverse healthcare settings.

## Figures and Tables

**Table 1:** List of papers included in the scoping review. For each paper, the following information is reported: the device used to show the digital information to the user, the sensors used to collect data to generate the augmented information, the pathology and the anatomical district the proposed system addressed, the number of participants the system was tested on, the assessment of acceptability and usability.

Author	AR/MR Visualization Device	AR/MR	Source of Information in AR/MR	Pathology	Anatomical District	Number of Participants	Acceptability and/or Usability Analysis
Ahn et al., 2017 [29]	HMD ^1^ ^(Epson Moverio)^	AR	•Accelerometer •Gyroscope	Parkinson	Lower-limb	P ^5^: 10	No
Aung et al., 2014 [30]	Monitor	AR	•Camera •EMG sensor	Stroke	Upper-limb	H ^6^: 7	No
Barioni et al., 2017 [31]	Monitor	AR	RGB-D camera	NSND ^3^	Upper-limb	H: 9	Yes
Bennour et al., 2018 [32]	Video Projector	AR	Infrared camera	NSND	Lower-limb	H: 10	No
Blomqvist et al., 2021 [33]	HMD ^(Microsoft HoloLens)^	AR	SLAM ^2^	Impaired balance	Whole body	P: 7	Yes
Boucher et al., 2013 [34]	HMD ^(VUZIX iWear)^	AR	RGB-D camera	Parkinson	Whole body	33 (H: 11, P: 22)	Yes
Burke et al., 2010 [35]	Monitor	AR	Camera	Stroke	Upper-limb	No info	No
Cavalcanti et al., 2019 [36]	Monitor	AR	RGB-D camera	NSND	Upper-limb	H: 45	Yes
Chen et al., 2011 [37]	Monitor	MR	•Infrared camera •Pressure sensor	Stroke	Upper-limb	H: 3	No
Colomer et al., 2016 [38]	Video Projector	AR	RGB-D camera	Stroke	Upper-limb	P: 30	Yes
Condino et al., 2019 [39]	HMD ^(Microsoft HoloLens)^	MR	SLAM	NSND	Upper-limb	H: 25	Yes
Da Gama et al., 2016 [40]	Monitor	AR	RGB-D camera	NSND	Upper-limb	33 (H: 22, P:11)	Yes
de Assis et al., 2016 [41]	Monitor	AR	•EMG sensor •Camera	Stroke	Upper-limb	P: 8	No
De Cecco et al., 2023 [42]	HMD ^(Microsoft HoloLens)^	MR	•RGB-D camera •Force platform •ECG sensor	NSND	Upper-limb	8 (H: 5, P: 3)	No
de Crignis et al., 2023 [43]	HMD ^(Microsoft HoloLens)^	AR	SLAM	Stroke	Upper-limb	P: 11	Yes
De Leon et al., 2014 [44]	Monitor	AR	RGB-D camera	Stroke	Upper-limb	H:4	Yes
Debarba et al., 2018 [45]	HMD ^(Microsoft HoloLens)^	AR	•SLAM •Infrared camera	NSND	Lower-limb	H: 5	Yes
Duff et al., 2012 [46]	Monitor	MR	Infrared camera	Stroke	Upper-limb	P:25	No
Enam et al., 2021 [47]	Video Projector	AR	Force platform	Stroke	Lower-limb	3 (H: 1, P: 2)	Yes
Escalona et al., 2020 [48]	Monitor	AR	RGB-D camera	NSND	Whole body	H: 10	Yes
Espay et al., 2010 [49]	HMD ^(N/A)^	AR	•Accelerometer •Force platform	Parkinson	Lower-limb	P: 13	No
Evans et al., 2022 [50]	HMD ^(Microsoft HoloLens)^	MR	SLAM	NSND	Lower-limb	H: 12	No
Everard, et al., 2024 [26]	Monitor	MR	•EMG sensor •Camera	PLP ^4^	Upper-limb	P: 81	Yes
Fang et al., 2023 [51]	Monitor	MR	RGB-D camera	Stroke	Upper-limb	P: 5	No
Franzò et al., 2023 [52]	HMD ^(Microsoft HoloLens)^	MR	SLAM	Cerebellar Ataxic	Upper-limb	H: 1	No
Franzo et al., 2023 [53]	HMD ^(Microsoft HoloLens)^	MR	SLAM	Cerebellar Ataxic	Upper-limb	No info	No
Garcia et al., 2014 [54]	Monitor	AR	RGB-D camera	Ankle sprain	Lower-limb	No info	No
Garcia Hernandez et al., 2023 [55]	HMD ^(Microsoft HoloLens)^	MR	SLAM	NSND	Upper-limb	H: 3	No
Gazzoni et al., 2021 [4]	HMD ^(Epson Moverio)^	AR	EMG sensor	NSND	Whole body	No info	No
Gmez-Portes et al., 2021 [56]	HMD ^(Microsoft HoloLens)^	MR	SLAM	Stroke	Upper-limb	H: 25	No
Guinet et al., 2022 [57]	HMD ^(Microsoft HoloLens)^	AR	SLAM	Cerebral Palsy	Lower-limb	P: 25	Yes
Gulcan et al., 2022 [58]	Video Projector	AR	Force platform	Parkinson	Lower-limb	P: 30	No
Ham et al., 2024 [59]	Monitor	MR	•Infrared camera •Camera	Stroke	Upper-limb	P: 21	No
Hazubski et al., 2020 [60]	HMD ^(Epson Moverio)^	AR	•Infrared camera •Accelerometer	Limb loss—Prosthesis control	Upper-limb	No info	No
He et al., 2018 [61]	Monitor	AR	•IMU sensor •Camera •LDR Sensor	NSND	Upper-limb	H: 5	No
Held et al., 2020 [62]	HMD ^(Microsoft HoloLens)^	AR	SLAM	Stroke	Lower-limb	P: 1	Yes
Hoda et al., 2014 [63]	Monitor	MR	•Infrared camera •Accelerometer	Stroke	Upper-limb	H: 6	Yes
Hossain et al., 2016 [64]	Monitor	AR	•Camera •Accelerometer •Vibrotactile actuators	Stroke	Upper-limb	36 (H: 25, P: 11)	Yes
Hunt et al., 2023 [65]	HMD ^(Custom made, VIVE Pro HTC)^	AR	•HTC Vive Tracker •EMG sensor •Camera	Limb loss—Prosthesis control	Upper-limb	H: 12	No
Im et al., 2015 [66]	Monitor	AR	RGB-D camera	NSND	Lower-limb	H: 18	Yes
Janssen et al., 2020 [67]	HMD ^(Microsoft HoloLens)^	MR	SLAM	Parkinson	Whole body	P: 16	Yes
Jeon et al., 2020 [68]	Monitor	AR	RGB-D camera	sarcopenia	Whole body	H: 27	Yes
Jin et al., 2019 [69]	HMD ^(Custom made)^	AR	•Force platform •RGB-D camera	Stroke	Lower-limb	H: 3	No
Jung et al., 2013 [70]	HMD ^(SVGA i-visor)^	AR	•EMG sensor •Electronic goniometer	Stroke	Upper-limb	P: 10	No
Karatsidis et al., 2018 [71]	HMD ^(Microsoft HoloLens)^	AR	•IMU sensor •SLAM	Knee osteoarthritis	Lower-limb	H: 11	No
Ko et al., 2021 [72]	HMD ^(Microsoft HoloLens)^	MR	SLAM	Stroke	Lower-limb	P: 9	No
Kong et al. [73]	Monitor	AR	RGB-D camera	Stroke	Whole body	10 (H: 8, P: 2)	Yes
Koroleva et al., 2021 [74]	HMD ^(Epson Moverio)^	AR	•Infrared camera •RGB-D camera	Stroke	Whole body	P: 50	No
Ku et al., 2019 [75]	Monitor	AR	RGB-D camera	NSND	Lower-limb	H: 34	No
Kuijpers et al., 2022 [76]	Video Projector	AR	Force platform	Developmental Coordination Disorder	Lower-limb	P: 27	Yes
Lancere et al., 2023 [77]	HMD ^(Microsoft HoloLens)^	MR	•EMG sensor •Respiratory Sensor	Limb loss—Breathing training	Deep core muscles	P: 13	Yes
Lee et al., 2019 [78]	HMD ^(Microsoft HoloLens)^	MR	•SLAM •Force platform	Impaired balance	Whole body	H: 8	Yes
Li et al., 2021 [79]	Monitor	AR	Camera	Stroke	Upper-limb	P: 30	Yes
Lim G et al., 2024 [80]	HMD ^(Meta Quest Pro)^	MR	•EMG sensor •RGB-D camera	Amputees—Prosthesis control	Lower-limb	15 (H: 5, P: 10)	No
Lim JY et al. [81]	Monitor	AR	RGB-D camera	Anterior Cruciate Ligament	Lower-limb	P: 28	Yes
Lin et al., 2011 [82]	Monitor	AR	RGB-D camera	NSND	Upper-limb	No info	No
Liu et al., 2017 [83]	Monitor	AR	Camera	NSND	Upper-limb	H: 20	Yes
Liu et al., 2024 [84]	HMD/Monitor ^(N/A)^	MR	•SLAM •RGB-D camera	Pelvic Floor Dysfunction	Lower-limb	P: 1	No
Luchetti et al., 2020 [85]	HMD ^(Microsoft HoloLens)^	AR	SLAM	NSND	Lower-limb	H: 27	Yes
Mahmood et al. [86]	HMD ^(Microsoft HoloLens)^	MR	SLAM	Parkinson	Upper-limb	31 (H: 22, P: 9)	Yes
Markovic et al., 2014 [87]	HMD ^(VUZIX iWear)^	AR	•EMG sensor •Camera	Amputees—Prosthesis control	Upper-limb	H: 13	No
McCarty, T et al., 2024 [88]	HMD ^(Microsoft HoloLens)^	MR	•IMU sensor •SLAM •RGB-D camera •Force platform •RGB-D camera	NSND	Whole body	No info	No
Miller et al., 2022 [89]	HMD ^(Microsoft HoloLens)^	AR	SLAM	NSND	Lower-limb	H: 8	No
Miller et al., 2024 [90]	HMD ^(Microsoft HoloLens)^	AR	SLAM	NSND	Lower-limb	H: 19	No
Mousavi Hondori et al., 2016 [91]	Video Projector	AR	•Camera	Stroke	Upper-limb	P: 18	No
Nam et al., 2022 [92]	HMD ^(Microsoft HoloLens)^	MR	SLAM	NSND	Upper-limb	H: 4	Yes
Nam et al., 2023 [93]	Monitor	AR	IMU sensor	Adolescent idiopathic scoliosis	Upper-limb	13 (H: 10, P: 3)	No
Nekar et al., 2023 [94]	HMD ^(Microsoft HoloLens)^	MR	•IMU sensor •EMG sensor •SLAM	NSND	Upper-limb	H: 32	Yes
Ortiz-Catalan et al., 2016 [95]	Monitor	AR	•Camera •EMG sensor	PLP	Upper-limb	P: 14	No
Pavlou et al., 2024 [28]	HMD/Video Projector ^(N/A)^	MR	•IMU sensor •Pressure-based insole •RGB-D camera	Impaired balance	Whole body	H: 120	Yes
Pezzera et al., 2020 [96]	HMD ^(Microsoft HoloLens)^	MR	•RGB-D camera •Force platform	NSND	Whole body	No info	No
Pillai et al., 2022 [97]	HMD ^(Microsoft HoloLens)^	MR	SLAM	NSND	Upper-limb	H: 10	Yes
Pinto-Fern’andez et al., 2023 [98]	HMD ^(Microsoft HoloLens)^	AR	IMU sensor	NSND	Lower-limb	H: 5	Yes
Pisano et al., 2024 [99]	Video Projector	AR	•Force platform •RGB-D camera	Parkinson	Lower-limb	P: 17	No
Prahm et al., 2022 [100]	HMD ^(Microsoft HoloLens)^	AR	•SLAM •EMG sensor	PLP	Upper-limb	No info	No
Retzinger et al., 2024 [101]	HMD ^(Magic Leap)^	AR	IMU sensor	Parkinson	Lower-limb	H: 20	Yes
Rizzi et al., 2023 [102]	HMD ^(Microsoft HoloLens)^	AR	•IMU sensor •Respiratory Sensor	NSND	Whole body	H: 10	Yes
Roumpi et al., 2022 [103]	HMD/Video Projector ^(N/A)^	AR	•IMU sensor •Pressure-based insole •RGB-D camera	Impaired balance	Whole body	H: 47	Yes
Scheermesser et al., 2024 [104]	HMD ^(Microsoft HoloLens)^	MR	SLAM	Stroke	Upper-limb	P: 15	Yes
Sekhavat et al., 2018 [105]	Video Projector	AR	RGB-D camera	NSND	Lower-limb	32 (H: 24, P: 8)	Yes
Sharma et al., 2018 [106]	HMD ^(Microsoft HoloLens)^	MR	•SLAM •EMG sensor	Limb loss—Prosthesis control	Upper-limb	H: 2	No
Shim et al., 2022 [107]	HMD ^(Microsoft HoloLens)^	MR	•EMG sensor •SLAM	Limb loss—Prosthesis control	Lower-limb	15 (H: 8, P: 7)	Yes
Shim et al., 2023 [25]	Monitor	AR	RGB-D camera	Knee osteoarthritis	Lower-limb	P: 56	Yes
Shim et al., 2023 [27]	Monitor	AR	RGB-D camera	Rotator cuff tear	Upper-limb	P: 115	Yes
Sousa et al., 2016 [108]	Video Projector	AR	Infrared camera	NSND	Upper-limb	H: 18	Yes
Tada et al., 2022 [109]	HMD ^(Microsoft HoloLens)^	MR	SLAM	NSND	Upper-limb	H: 7	No
Tada et al., 2024 [110]	HMD ^(Microsoft HoloLens)^	MR	SLAM	NSND	Upper-limb	H: 10	Yes
Tan et al., 2024 [111]	HMD ^(Microsoft HoloLens)^	MR	•RGB-D camera •SLAM •IMU sensor	NSND	Lower-limb	H: 10	Yes
Tanda et al., 2024 [112]	HMD ^(Microsoft HoloLens)^	MR	•SLAM •Camera	Multiple Sclerosis	Upper-limb	H: 13	Yes
Thinh et al., 2021 [113]	Monitor	AR	•Camera •Torque sensor	Stroke	Whole body	P: 10	Yes
Thøgersen et al., 2020 [114]	HMD ^(HTC Vive)^	AR	•HTC Vive Tracker •EMG sensor	PLP	Upper-limb	P: 7	No
Timmermans et al., 2021 [115]	Video Projector	AR	•Force platform •RGB-D camera	Stroke	Lower-limb	P: 33	Yes
Trojan et al., 2014 [116]	HMD/Monitor ^(eMagin)^	AR	•Camera •Infrared camera	NSND	Upper-limb	H: 7	No
Tykhyi et al., 2024 [117]	HMD ^(Magic leap)^	AR	SLAM	NSND	Upper-limb	P: 1	No
Vaida et al., 2024 [118]	HMD ^(Microsoft HoloLens)^	AR	•SLAM •Electronic goniometer	NSND	Lower-limb	H:12	Yes
van de Venis et al., 2023 [119]	Video Projector	AR	Force platform	HSP ^7^	Lower-limb	P: 36	No
Viglialoro et al., 2023 [120]	Video Projector	AR	Infrared camera	NSND	Upper-limb	H: 16	Yes
Wang et al., 2020 [121]	HMD ^(HTC Vive)^	AR	•HTC Vive Tracker •Camera	Parkinson	Lower-limb	P: 5	Yes
Wang et al., 2022 [122]	Video Projector	AR	Force platform	Parkinson	Lower-limb	P: 52	No
Wang et al., 2023 [123]	HMD ^(Oculus Quest)^	AR	Infrared camera	Tremor	Whole body	H: 13	Yes
Wang et al., 2024 [124]	HMD ^(Microsoft HoloLens)^	MR	SLAM	Stroke	Upper-limb	P:12	Yes
Wenk et al., 2019 [125]	HMD/Monitor ^(HTC Vive)^	AR	HTC Vive Tracker	NSND	Upper-limb	H: 20	No
Yang et al., 2022 [126]	Monitor	AR	RGB-D camera	Stroke	Whole body	P: 39	Yes
Yu et al., 2023 [127]	Monitor	AR	RGB-D camera	Knee osteoarthritis	Lower-limb	P: 24	No
Zhang et al., 2010 [128]	Monitor	AR	•RGB-D camera •Flex sensor	Stroke	Upper-limb	No info	No

^1^ HMD: Head Mounted Display. ^2^ SLAM: Simultaneous Localization and Mapping. ^3^ NSND: Not Specified Neuromuscular Disorders. ^4^ PLP: Phantom Limb Pain. ^5^ P: Patients. ^6^ H: Healthy participants. ^7^ HSP: Hereditary Spastic Paraplegia.

## Data Availability

No new data were created or analyzed in this study. Data sharing does not apply to this article.

## Abbreviations

The following abbreviations are used in this manuscript:

AR	Augmented reality
EMG	Electromyography
FOG	Freezing of gait
HMD	Head-mounted display
IMU	Inertial measurement unit
MR	Mixed reality
NSND	Not Specified Neuromuscular Disorders
PLP	Phantom limb pain
PRISMA-ScR	Systematic reviews and meta-analyses extension for scoping reviews
SLAM	Simultaneous localization and mapping
SUS	System usability scale
VR	Virtual reality
XR	Extended reality

## Appendix A

The search was performed using the following queries:

Scopus:

TITLE-ABS-KEY ((“mixed-reality” OR “augmented reality” OR “Hololens”) AND (“rehabilitation”)) AND PUBYEAR > 2010 AND (LIMIT-TO (DOCTYPE, “cp”) OR LIMIT-TO (DOCTYPE, “ar”) OR LIMIT-TO (DOCTYPE, “re”)) AND (LIMIT-TO (LANGUAGE, “English”)) AND (LIMIT-TO (SRCTYPE, “j”) OR LIMIT-TO (SRCTYPE, “p”)).

PubMed:

((“augmented reality” [MeSH Terms] OR (“augmented” [All Fields] AND “reality” [All Fields]) OR “augmented reality” [All Fields] OR (“mixed” [All Fields] AND “reality” [All Fields]) OR “mixed reality” [All Fields] OR (“augmented reality” [MeSH Terms] OR (“augmented” [All Fields] AND “reality” [All Fields]) OR “augmented reality” [All Fields]) OR “Hololens” [All Fields]) AND (“rehabilitant” [All Fields] OR “rehabilitants” [All Fields] OR “rehabilitate” [All Fields] OR “rehabilitated” [All Fields] OR “rehabilitates” [All Fields] OR “rehabilitating” [All Fields] OR “rehabilitation” [MeSH Terms] OR “rehabilitation” [All Fields] OR “rehabilitations” [All Fields] OR “rehabilitative” [All Fields] OR “rehabilitation” [MeSH Subheading] OR “rehabilitation s” [All Fields] OR “rehabilitational” [All Fields] OR “rehabilitator” [All Fields] OR “rehabilitators” [All Fields])) AND ((english [Filter]) AND (2010:2024[pdat]))
